# USP14 inhibition promotes recovery by protecting BBB integrity and attenuating neuroinflammation in MCAO mice

**DOI:** 10.1111/cns.14292

**Published:** 2023-06-02

**Authors:** Wenzhong Hou, Jianping Yao, Junjie Liu, Xiaohong Lin, JueXian Wei, Xiaofan Yin, Hongbiao Huang, Xiaohui Chen, Guo‐Yuan Yang, Xiaosong He

**Affiliations:** ^1^ Department of Cerebrovascular Disease, The Sixth Affiliated Hospital of Guangzhou Medical University Qingyuan People's Hospital Qianyuan China; ^2^ Department of Anatomy, School of Basic Medical Science Guangzhou Medical University Guangzhou China; ^3^ Department of Emergency The Second Affiliated Hospital of Guangzhou Medical University Guangzhou China; ^4^ Department of Pathophysiology, School of Basic Medical Sciences Guangzhou Medical University Guangzhou China; ^5^ Neuroscience and Neuroengineering Center Shanghai Jiao Tong University School of Biomedical Engineering Shanghai China; ^6^ Department of Neurology, Institute of Neuroscience, Key Laboratory of Neurogenetics and Channelopathies of Guangdong Province and the Ministry of Education of China, The Second Affiliated Hospital Guangzhou Medical University Guangzhou China; ^7^ School of Basic Medical Sciences Guangzhou Medical University Guangzhou China

**Keywords:** behavior recovery, blood–brain barrier, ischemic stroke, neuroinflammation, USP14

## Abstract

**Aim:**

Blood–brain barrier (BBB) dysfunction is one of the hallmarks of ischemic stroke. USP14 has been reported to play a detrimental role in ischemic brain injury. However, the role of USP14 in BBB dysfunction after ischemic stroke is unclear.

**Methods:**

In this study, we tested the role of USP14 in disrupting BBB integrity after ischemic stroke. The USP14‐specific inhibitor IU1 was injected into middle cerebral artery occlusion (MCAO) mice once a day. The Evans blue (EB) assay and IgG staining were used to assess BBB leakage 3 days after MCAO. FITC‐detran test was slected to examine the BBB leakage in vitro. Behavior tests were conducted to evaluate recovery from ischemic stroke.

**Results:**

Middle cerebral artery occlusion increased endothelial cell USP14 expression in the brain. Furthermore, the EB assay and IgG staining showed that USP14 inhibition through IU1 injection protected against BBB leakage after MCAO. Analysis of protein expression revealed a reduction in the inflammatory response and chemokine release after IU1 treatment. In addition, IU1 treatment was found to rescue neuronal loss resulting from ischemic stroke. Behavior tests showed a positive effect of IU1 in attenuating brain injury and improving motor function recovery. In vitro study showed that IU1 treatment could alleviate endothelial cell leakage induced by OGD in cultured bend.3 cells through modulating ZO‐1 expression.

**Conclusions:**

Our results demonstrate a role for USP14 in disrupting the integrity of the BBB and promoting neuroinflammation after MCAO.

## Graphical abstract

Ischemic stroke induced higher endothelial deubiquitinating enzymes Usp14 expression in the brain. Inhibition Usp14 expression through specific inhibitor IU1 injection increased ZO‐1 expression, attenuated blood–brain barrier leakage, inhibited neuroinflammation, reduced neuronal loss, and promoted neural behavior recovery after middle cerebral artery occlusion.

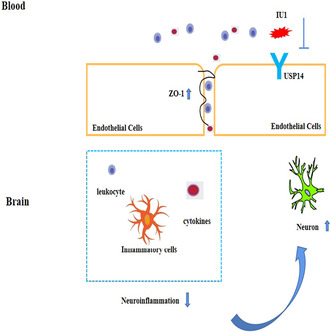

## 1 INTRODUCTION

Ischemic stroke is a vascular brain disease caused by the occlusion of blood vessels, and it leads to severe motor dysfunction and mortality and imposes an economic burden on society. Currently, in clinical practice, there are no treatments for acute ischemic stroke except for rtPA and thrombectomy.^1^, ^2^, ^3^ Despite the great progress in endovascular therapy for stroke in the past several decades, the overall therapeutic efficiency remains low.^4^, ^5^ Hence, stroke is still a substantial public health concern for which novel treatment approaches are greatly needed. One of the clinical pathological characteristics of ischemic stroke is blood–brain barrier (BBB) dysfunction. BBB disruption results in brain edema and invasion of substances in the blood into the brain parenchyma, which contributes to neuroinflammation and further neuronal cell death.^6^ Clearly, strategies targeted to preserve BBB integrity may effectively prevent disability and enhance recovery after ischemic stroke.

Normal BBB structure is important for maintaining the microenvironment of the brain.^7^ After stroke, a series of proteins involved in maintaining BBB structure are up‐ or downregulated, suggesting that protein clearance pathways may contribute to BBB dysfunction.^8^ Among those proteins, tight junction proteins are of great importance in maintaining BBB integrity. Tight junction proteins located on the surface of endothelial cells are highly degraded after ischemic stroke. Preventing tight junction protein degradation helps to maintain the structural integrity of the BBB and attenuate neuroinflammation after ischemic stroke.^9^, ^10^ Several mechanisms, such as ROS‐ and MMP‐mediated protein degradation, have been reported to contribute to tight junction alterations and subsequently to the disruption of BBB integrity.^11^, ^12^ Recently, studies have also shown that the lysosome‐dependent protein degradation pathway is involved in tight junction alterations after ischemic stroke. For example, Zhang et al. reported that autophagy participated in CLN5 degradation and disruption of BBB integrity after ischemic stroke.^13^ In addition to autophagy, tight junction proteins can be degraded through another typical protein degradation pathway, the ubiquitin–proteasome system (UPS).^14^ However, little is known about the role of deubiquitinating enzymes (DUBs) in BBB dysfunction after ischemic stroke.

Deubiquitinating enzymes are a large set of proteases that can remove ubiquitin protein from target proteins to reverse ubiquitination. USP14 is a well‐studied DUB belonging to the UPS family that plays dual roles in regulating protein degradation.^15^ Dysregulated USP14 expression leads to various pathologies, such as inflammation, apoptosis, and even ferroptosis, by either promoting or inhibiting protein degradation, which results in cancer, neurobiological diseases, and atherosclerosis‐related diseases.^16^, ^17^, ^18^, ^19^, ^20^ USP14 was first reported to be expressed on the neurons and to negatively regulate neuronal survival in the brain.^21^, ^22^ According to data from single‐cell sequencing of vascular cells, USP14 is also highly expressed on brain endothelial cells.^23^, ^24^ However, the effect of USP14 on endothelial tight junction proteins and BBB function, especially under disease conditions, has not been well studied.

In the present study, using a mouse model of middle cerebral artery occlusion (MCAO), we examined the effect of the USP14 inhibitor IU1 on BBB function after ischemic stroke in mice. We found that treatment with IU1 once a day effectively ameliorated ischemic injury and improved behavior by preventing ZO‐1 degradation and protecting BBB integrity. These results provide insight into the mechanism of BBB dysfunction after ischemic stroke and improve our understanding of the role of USP14 in stroke pathology.

## 2 METHODS AND MATERIALS

### 2.1 Animals

Male adult ICR mice (aged 8–10 weeks, 25–28 g) obtained from Guangdong Animal Center were used in this study. The animals were housed under standard conditions. All animal handling was performed in a manner to minimize suffering. The animal procedures were performed following the Guide for the Care and Use of Laboratory Animals of the US National Institutes of Health and reported in accordance with the ARRIVE guidelines.^25^ All experimental protocols were approved by the Guangzhou Medical University Institutional Animal Care and Use Committee.

### 2.2 Mouse model of MCAO and drug treatment

As previously reported, mice were fully anesthetized by intraperitoneal injection of 1% sodium pentobarbital.^26^ Then, the left external carotid artery (ECA), internal carotid artery, and common carotid artery (CCA) were exposed under an operating microscope, and a 6‐0 suture (Ruiwode) was inserted into the origin of the middle cerebral artery (MCA) to occlude blood flow. Body temperature was maintained at 37 ± 0.5°C with a temperature‐controlled heating pad during surgery. Reperfusion was performed by withdrawing the suture 60 min later. Cerebral blood flow (CBF) was tested using laser–Doppler flowmetry before and after surgery. CBF immediately decreased to 10% of the baseline level after suture insertion and returned to 70% of the baseline level after suture withdrawal, indicating successful MCAO and reperfusion, respectively. Mice in which MCAO and reperfusion were not successful were excluded from further experimentation. Mice with apparent dots of blood in brain tissue during sectioning were also excluded from data analysis. All mice were randomly divided into three groups: the sham group, which included mice that underwent the same surgery as the other mice excluding suture insertion; the IU1‐treated group, which included mice that underwent MCAO surgery and were treated with IU1; and the control group, which included mice that underwent MCAO surgery and were treated with vehicle (saline). After reperfusion, 400 μg/kg body weight IU1 (MedChemExpress) was intraperitoneally injected as reported previously.^27^ The first dose of IU1 was intraperitoneally injected right after reperfusion, and other two doses were given 24 and 48 h post ischemic stroke respectively.

### 2.3 Behavior tests

#### 2.3.1 Bederson scores and modified neurological severity scores

Bederson scores and modified neurological severity scores (mNSSs) were determined by a researcher who was blinded to the experimental design. Bederson scores ranged from 0 to 5, and the scoring criteria were modified for use in mice.^28^ Mice that were disabled in moving were excluded with further analysis in behavior test.

Modified neurological severity scores ranged from 0 to 14 and were used to assess overall behavioral performance, including motor function, reflexes, and balance.^29^

#### 2.3.2 Open field test

The mice were placed into the center of a 50 × 50 × 50 cm arena and were allowed to explore freely for 5 min. Each 5 min trial was recorded by a digital camera connected to a computer equipped with the EthoVision XT video tracking system (Noldus). The total distance traveled and speed were calculated for each trial.

#### 2.3.3 Rotarod test

Mice were placed on an accelerating rotarod device (Zhenhua), and rod was slowly accelerated from 5 to 80 rpm within 5 min. The mice were placed on the rod, and the timers were started with acceleration and stopped when the mice fell off. The time spent on the rod was recorded, and the average of three values was calculated. A longer time spent on the rod indicated better motor coordination and balance.

### 2.4 Immunohistochemistry and IgG staining

Brains were quickly frozen with isopentane and sliced on a cryostat (Leica) at a thickness of 20 μm. For immunofluorescence staining, the sections were fixed with 4% paraformaldehyde, rinsed 3 times with PBS for 5 min, blocked with 5% normal donkey serum for 60 min, and incubated with primary antibodies against GFAP (1:600; Millipore), IBA1 (1:500; WAKO), NeuN (1:1000; Millipore), MPO (1:200; Abcam), USP14 (1:100; Abcam), ZO‐1 (1:200; Invitrogen), and CD31 (1:200; R&D) at 4°C for more than 24 h. The next day, the sections were incubated with corresponding secondary antibodies for 120 min. For IgG staining, the sections were stained with avidin–biotin complex (Vector Labs), 3,3′‐Diaminobenzidine (DAB) was added for the visualization of IgG staining. For terminal deoxynucleotidyl transferase‐mediated dUTP nick‐end labeling (TUNEL) and NeuN double staining, the sections were first treated with TUNEL kit reagents at 37°C for 30 min and then stained with a NeuN antibody according to a routine protocol. Two sections from each mouse were evaluated, and three fields per section were sampled. All immunostaining data were analyzed by a blinded investigator. Immunofluorescence images were taken by a confocal microscope (Leica), and other images were taken by a regular digital microscope (Nikon).

### 2.5 Brain infarct volume assessment

Twelve 20 μm thick serial frozen sections (240 μm intervals) were selected and stained with 0.1% cresyl violet. The infarct border was carefully determined, and the infarct area was evaluated using ImageJ software as previously described. The brain infarct volume was determined by a previously reported.^30^

### 2.6 Western blot analysis

Western blot analysis was conducted as regularly.^31^ Protein samples were extracted from bend.3 cells using RIPA lysis buffer. For the in vivo study, the brain was quickly removed to a cooled brain mold, and then cut into four coronal sections with 2 mm apart; the second section including ischemic core was selected for protein extraction. Tissues were homogenized with by ultrasonication on ice and then centrifuged. All the protein concentrations were quantified by a kit (Beyotime, P0012). Equal amounts of protein were loaded on SDS‐PAGE gels and transferred to PVDF membranes. For immunoblotting, the membrane was first blocked with 5% nonfat milk and then incubated with primary antibody at 4°C overnight. The primary antibodies used were as follows: anti‐ZO‐1 (1:1000), anti‐GFAP (1:1000), anti‐MPO (1:1000), anti‐USP14 (1:500), and anti‐GAPDH (1:2000). Semiquantitative analysis of the chemiluminescence signal was performed, and the gray values of all bands were standardized to that of the GAPDH band. Immunoblot band intensities were quantified using ImageJ software.

### 2.7 ELISA

ELISA was performed to test the protein levels of interleukin‐1β (IL‐1β) (EK0394; Boshide), tumor necrosis factor‐a (TNF‐a) (EK0527β; Boshide), and interleukin‐6 (IL6) (EK0411; Boshide) in animals according to the manufacturer's instructions. Briefly, protein was collected and diluted with lysis buffer, and the concentration of protein was quantified according to a standard curve. The results are expressed as picograms per milligram protein.

### 2.8 Cell culture and OGD

Bend.3 endothelial cell was ordered from Procell (cat. CL‐0598) and maintained in Dulbecco's Modified Eagle Medium (C11995500BT; Gibco) with 15% FBS (Gibco), penicillin (100 U/mL) and streptomycin (100 mg/mL). All the cells were incubated in 5% CO_2_ incubator at 37°C. For OGD, the medium was replaced with DMEM without glucose and 8% FBS. Then, cultures were transferred to an anaerobic chamber infused with a gas mixture containing 5% CO_2_, 95% N_2_. After incubating for 8 h, cells were further cultured in Eagle supplemented with 15% FBS and glucose under normal conditions for another 4 h with or without IU1.

### 2.9 In vitro FITC‐dextran transwell assay

Bend.3 cells were seeded on the top chamber of transwell inserts (Corning, 3470). After exposing to OGD and IU1 treatment, 300 μg/mL FITC‐dextran (Sigma, 90718) was added to the top chamber. Fifteen minutes later, samples were collected from the bottom chamber for further analysis. Then, the fluorescence intensity was analyzed with a fluorometer at an excitation of 490 nm and an emission of 530 nm.

### 2.10 CCK‐8 assay

Bend.3 cells were cultured in 96 plate, after reaching to 90% confluence, IU1 was added into the culture medium. Twelve hours after IU1 treatment, 10 μL CCK‐8 solution (Dojindo, CK04‐500) was added. The absorbance (OD) value was detected at 450 nm 2 h later.

### 2.11 In vitro immunofluorenscence

Bend.3 cells were seeded on coverslips. After reaching to 80% confluence, cells were exposed to OGD with IU1 treatment. Then, cells were fixed with 4% polyoxymethylene. After washing with PBS, all other procedures such as blocking and antibody incubating were conduced similar to brain section immunostaining.

### 2.12 Evans blue assay

Mice were injected with EB dye solution (1% EB dye in saline, 4 mL/kg) through the tail vein and sacrificed after 2 h of EB circulation. Each brain hemisphere was weighed, and EB was extracted by incubating tissues with 50% TCA solution and centrifugation at 12,000 *g* for 30 min. The level of EB was quantified at 610 nm using a spectrophotometer.

### 2.13 Statistical analysis

Statistical analysis was carried out with Prism GraphPad 8 (GraphPad).

The Shapiro–Wilk test or D'Agostino and Pearson normality test was used to determine the normality of the data. All results are expressed as the mean ± SD. One‐way or two‐way ANOVA followed by Bonferroni's post hoc multiple comparisons test (normal distribution) or the Kruskal–Wallis test followed by Dunn's post hoc test (nonnormal distribution) was used for the analysis of multiple groups. Comparisons between two groups were made by two‐tailed Student's *t* test (normal distribution) or the Mann–Whitney test (non‐normal distribution). *p* < 0.05 was considered statistically significant. All n and *p* values and the statistical tests used are provided in the figure legends and/or results section.

## 3 RESULTS

### 3.1 USP14 expression was increased in brain endothelial cells after MCAO

**FIGURE 1:**
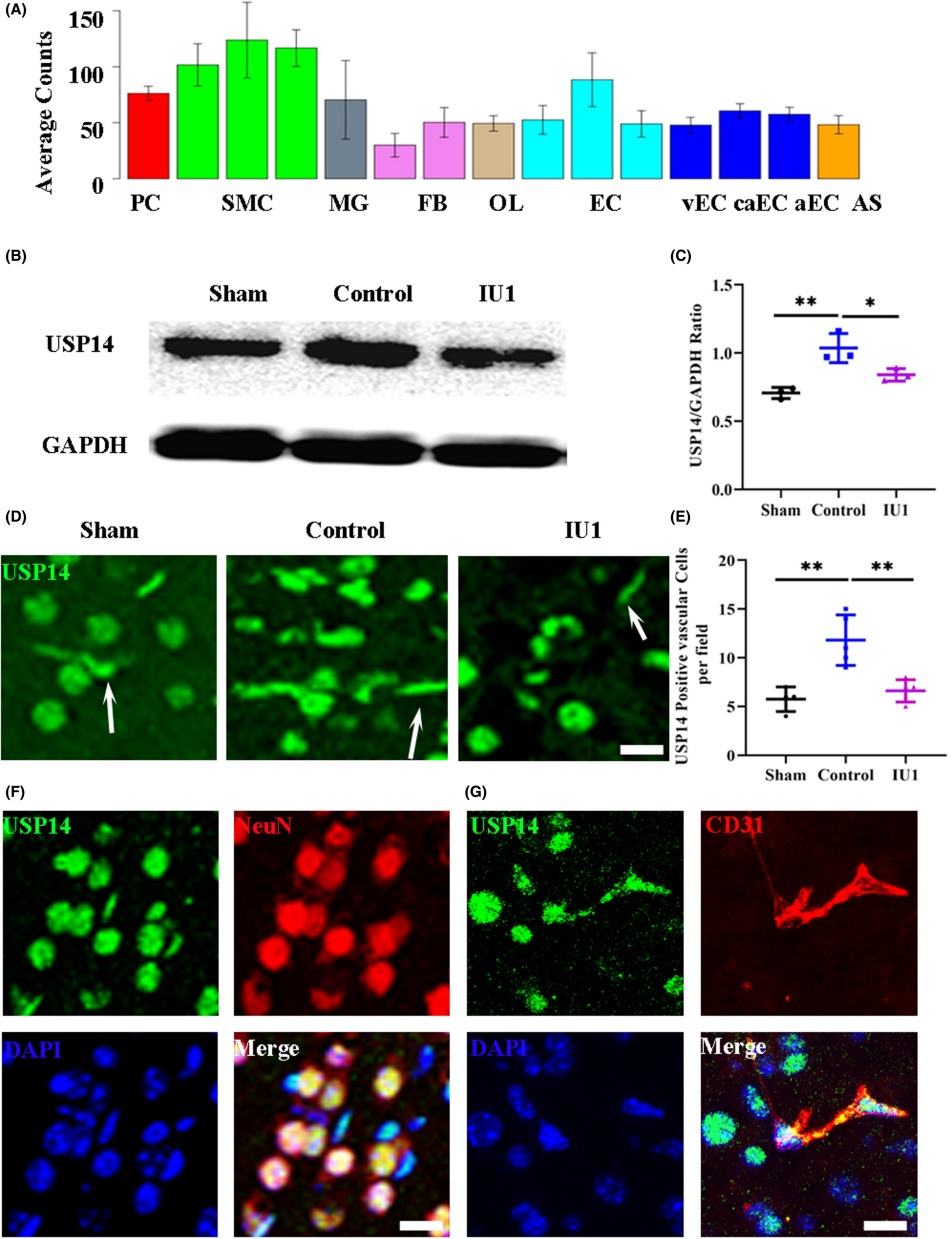
USP14 expression in the normal and ischemic brain. (A) Histogram showing the USP14 read counts from single‐cell sequencing of the brain. aEC, artery endothelial cells; AS, astrocytes; caEC, capillary endothelial cells; EC, endothelial cells; FG, fibroblasts; MG, microglial cells; OL, oligodendrocytes; PC, pericytes; SMC, smooth muscle cells; vEC, vein endothelial cells. (B) Western blot results showing USP14 expression in the brains of sham mice, ischemia model mice, and IU1‐treated ischemia model mice. (C) Quantification of the Western blot data. Note the increase in USP14 expression after MCAO and decrease in USP14 expression after IU1 treatment. *n* = 3 in each group; **p < 0.01, *p < 0.05. (D) Confocal images of immunostaining for USP14 expression in each group. The arrowheads indicate vascular‐like USP14‐positive cells; bar = 20 μm. (E) Quantification of USP14‐positive vascular cells; **p < 0.01 using one‐way ANOVA followed by Bonferroni's post hoc test; *n* = 4–5 in each group. (F) Double immunostaining showing that USP14 (green) colocalized with NeuN (red). (G) Typical confocal images showing that USP14 (green) was expressed on CD31‐positive endothelial cells (red). Bar = 20 μm.

We first obtained access to an open‐access single‐cell sequencing database from the Betsholtz laboratory (http://betsholtzlab.org/VascularSingleCells/database.html) to verify USP14 expression in the brain vascular cells.^23^, ^24^ The results showed that USP14 is expressed on most vascular cells, including smooth muscle cells, endothelial cells, pericytes, and even fibroblasts (Figure 1A). We then performed Western blotting to quantify USP14 expression at the protein level in normal and ischemic mouse brains. The results showed that ischemic stroke induced higher USP14 expression in the brain and that IU1 injection downregulated USP14 expression 3 days after MCAO (Figure 1B,C, *p* = 0.0039 and *p* = 0.0448). We also performed immunostaining to evaluate the location of USP14 in the brain. In sham mice, most USP14‐positive cells showed neuronal morphology, and some of them showed typical blood vessel morphology. Interestingly, there were many more USP14‐positive vascular‐like cells in MCAO mice than in sham mice and IU1‐treated MCAO mice (Figure 1D,E, *p* = 0.0013 and *p* = 0.0027). Then, using NeuN to label neurons and CD31 to label endothelial cells, we found that USP14 was expressed on the neurons (Figure 1E) and endothelial cells (Figure 1F) after ischemic stroke. These data suggest that endothelial cells are one of the sources of USP14 in the mouse brain, especially after brain ischemia.

### 3.2 IU1 protected BBB integrity and reduced leakage after MCAO

**FIGURE 2:**
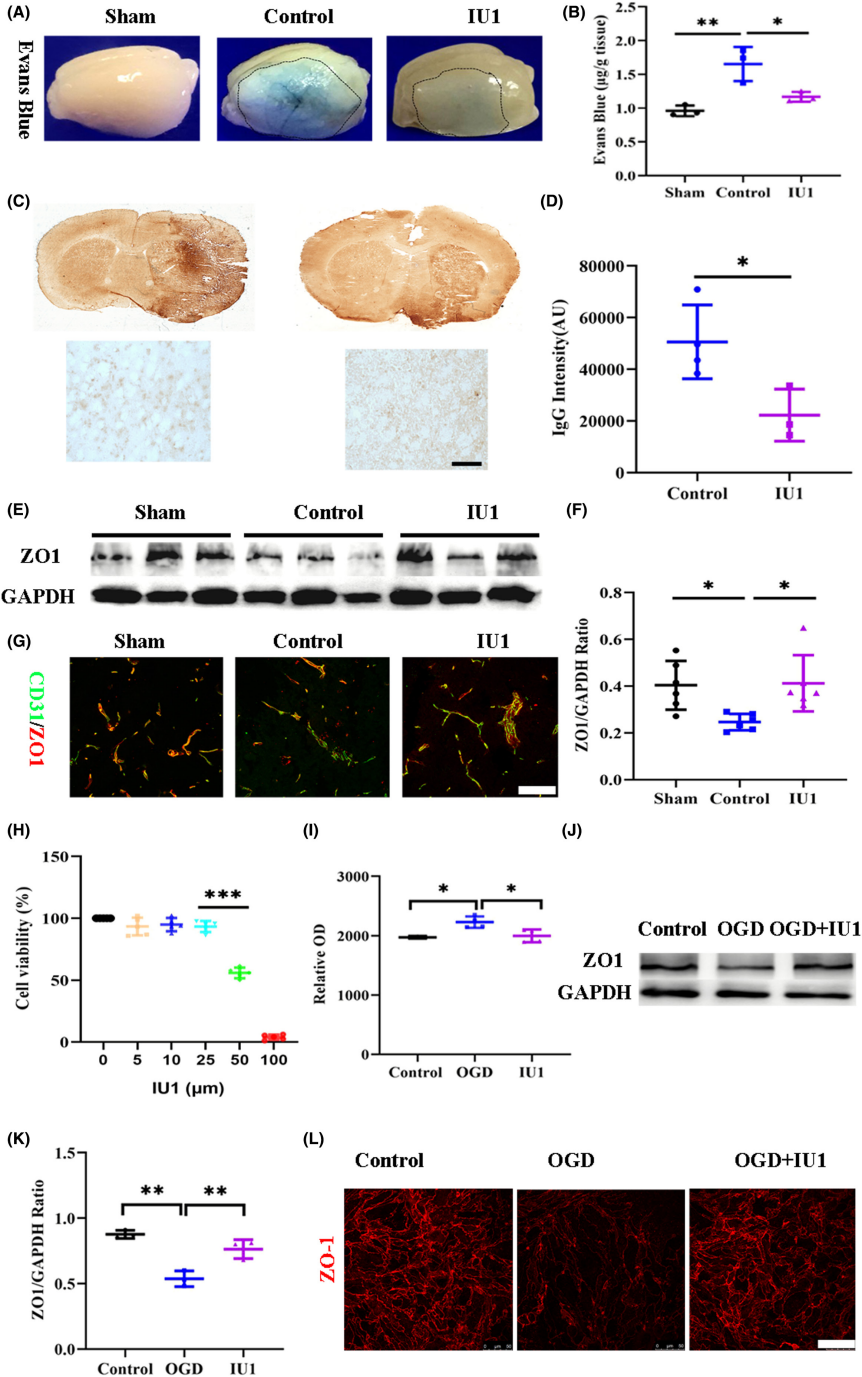
Effect of IU1 on blood–brain barrier integrity 3 days after middle cerebral artery occlusion (MCAO). (A) Typical images showing WB extravasation in the cortex 3 days after MCAO in each group. A dotted line indicates the area of WB leakage. (B) Bar graph showing quantitative EB leakage data; **p < 0.01, *p < 0.05 using one‐way ANOVA followed by Bonferroni's post hoc test. (C) Images of immunostained whole brain sections from control and IU1‐treated mice showing IgG leakage 3 days after MCAO. (D) Bar graph showing the intensity of IgG staining; *p < 0.05 using two‐tailed unpaired *t* test; *n* = 4 in the control group and *n* = 3 in the IU1 group; bar = 10 μm. (E) Western blot analysis of ZO‐1 expression in cortical tissue from each group 3 days after MCAO. (F) Quantification of the Western blot results; *p < 0.05 using one‐way ANOVA followed by Bonferroni's post hoc test; *n* = 6 in each group. (G) Typical confocal images showing ZO‐1 expression on CD31‐positive endothelial cells 3 days after MCAO; bar = 50 μm. (H) Histagram showed the cell viability after treating with IU1, ***p < 0.001 using one‐way ANOVA followed by Bonferroni's post hoc test; *n* = 5 in each group. (I) Histogram showed the endothelial cell leakage examined by FITC‐detran diffusion test; *p < 0.05 using one‐way ANOVA followed by Bonferroni's post hoc test; *n* = 3–4 in each group. (J) Western blot analysis of ZO‐1 expression in cultured bend.3 cells exposing to OGD and IU1 treatment. (K) Quantification of the Western blot results; **p < 0.01 using one‐way ANOVA followed by Bonferroni's post hoc test; *n* = 3 in each group. (L) Typical images showing ZO‐1 expression in different groups.

The increase in endothelial cell USP14 expression implies a potential role for USP14 in regulating the function of endothelial cells. Given the central role of endothelial cells in regulating BBB structure, we attempted to clarify the role of USP14 in BBB dysfunction after ischemic stroke. A specific USP14 inhibitor, IU1, was intravenously injected once a day after MCAO as previously reported. We first performed the Evans blue (EB) assay to assay EB extravasation in the ischemic brain. As expected, EB extravasation and accumulation were not observed in the sham mice. After MCAO, EB passed through the BBB and accumulated in the injured cortex (Figure 2A). However, less EB accumulation was observed on the surface of the brain after IU1 treatment. The quantitative data showed that EB extravasation was significantly reduced after IU1 treatment at 3 days after MCAO (Figure 2B, *p* = 0.0051 and *p* = 0.0278). We also used IgG staining to test BBB leakage. The IgG intensity was increased on the lesion side after MCAO but was significantly reduced after IU1 treatment (Figure 2C,D, *p* = 0.0338). We then assessed the expression of Zonula occludens 1 (ZO‐1) to further test whether IU1 treatment protected the structural integrity of the BBB. The Western blotting results showed that ZO‐1 expression after IU1 treatment was higher than that in the control mice (Figure 2E,F, *p* = 0.0335 and *p* = 0.0244). ZO‐1 was expressed in the endothelial cells in the sham‐operated mice. There were fewer ZO‐1/CD31‐positive cells in the control mice than in the sham mice. After IU1 treatment, more ZO‐1‐positive endothelial cells were observed in the brain (Figure 2G). To futher confirm the role of USP14 on the endothelial cells, we cultured Bend.3 cells exposing to OGD to mimic ischemic condition. The CCK‐8 assay showed that a lower concentration of IU1 (5, 10, and 25 μM) showed no toxicity to the cells (Figure 2H). Therefore, we chose 25 μM treatment as the optimal concentration of IU1 for subsequent in vitro experiments. The results from FITC‐detran diffusion test examining the intergrity of endothelial cells showed a significantly effect of IU1 in protecting BBB integrity (Figure 2I). Further Western blot (Figure 2J–L) and immunofluorenscence results confirmed the role of IU1 in preventing ZO‐1 alternation induced by OGD. These results suggest that IU1 protects the structural integrity of the BBB by preventing ZO‐1 degradation.

### 3.3 IU1 inhibited the inflammatory cell response and attenuated neuroinflammation after MCAO

**FIGURE 3:**
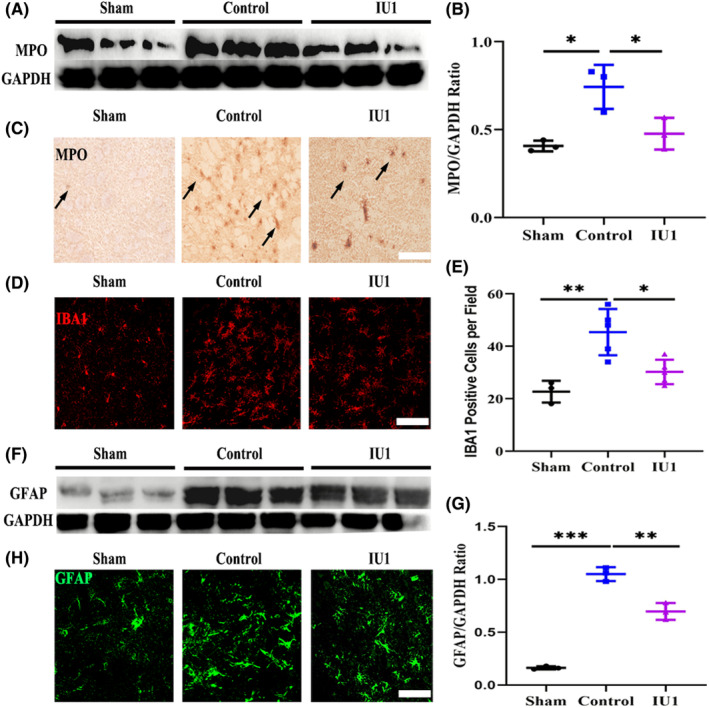
USP14 inhibition attenuated the inflammatory cell response in the brain. (A) Western blot analysis of myeloperoxidase (MPO) expression in the cortical tissue from each group 3 days after middle cerebral artery occlusion (MCAO). (B) Quantification of the Western blot results; *p < 0.05 using one‐way ANOVA followed by Bonferroni's post hoc test; *n* = 3 in each group. (C) Typical images showing MPO‐positive cells in the brain. The arrowheads indicate MPO‐positive cells, an increase in the number of MPO‐positive cells in the control group, and a decrease in the number of MPO‐positive cells in the IU1‐treated mouse brain. (D) Immunofluorescence images showing IBA1‐positive microglial cells in the mouse brains. (E) Bar graph showing the quantification of microglial cells; **p < 0.01, *p < 0.05 using one‐way ANOVA followed by Bonferroni's post hoc test; *n* = 3 in the sham group, *n* = 5 in the control group and *n* = 5 in the IU1 group. (F) Western blot analysis of GFAP expression in the cortical tissue from each group 3 days after MCAO. (G) Quantification of the Western blot results; ***p < 0.01, **p < 0.01 using one‐way ANOVA followed by Bonferroni post hoc test; *n* = 3 in each group. (H) Typical confocal images showing GFAP‐positive astrocytes in the brain in each group.

Leukocytes migrate to and infiltrate the brain parenchyma after BBB opening following ischemic stroke. Given the role of IU1 in protecting the structural integrity of the BBB, we further used myeloperoxidase (MPO) to test whether IU1 inhibited the infiltration of leukocytes. The Western blotting results showed that MPO expression was downregulated in IU1‐treated MCAO mice compared with control mice (Figure 3A,B, *p* = 0.0117 and *p* = 0.0341). Immunostaining showed that leukocytes accumulated in the injury site in MCAO mice, but there were fewer MPO‐positive cells in the IU1‐treated mice (Figure 3C). We also tested the resident inflammatory cell response in the brain after ischemic stroke. Using ionized calcium‐binding adapter molecule 1 (IBA1) to mark microglial cells, we noticed that microglial morphology was obviously changed after MCAO and that microglial cells exhibited enlarged cell bodies, which is a typical manifestation of microglial cell activation (Figure 3D). Quantitative analysis of microglial cell density showed that the number of microglial cells in the brains of MCAO mice was reduced after IU1 treatment (Figure 3E, *p* = 0.0024 and *p* = 0.0133). Then, using glial fibrillary acidic protein (GFAP) as a marker of activated astrocytes, we noticed that astrocyte activation was also reduced in the IU1‐treated mice compared with the control mice, as shown by Western blotting (Figure 3F,G, *p* = 0.00001 and *p* = 0.0011) and immunostaining (Figure 3H). These results suggest that IU1 inhibited the inflammatory cell response in the brain 3 days after MCAO.

**FIGURE 4:**
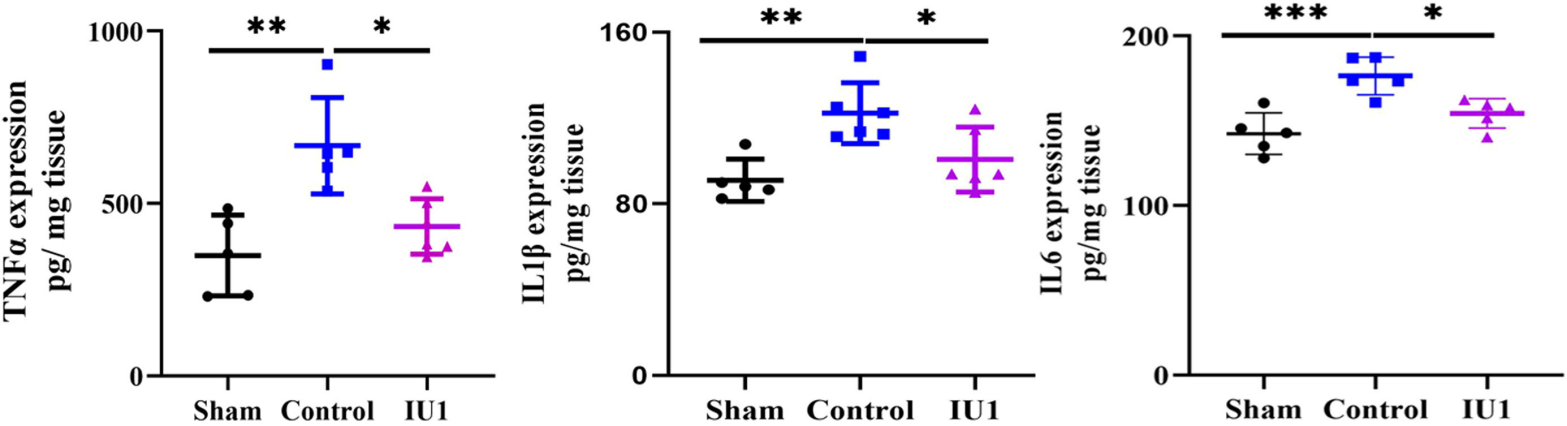
USP14 inhibition inhibited inflammatory cytokine release in the brain. Bar graph showing the protein levels of the inflammatory cytokines TNFα (left panel), IL‐1β (middle panel) and IL6 (right panel) in the brain 3 days after middle cerebral artery occlusion, as tested by ELISA. ***p < 0.001, **p < 0.01, *p < 0.05 using one‐way ANOVA followed by Bonferroni's post hoc test; *n* = 5–6 in each group.

Leukocytes and even resident glial cells are the major sources of chemokines and cytokines, which initiate neuroinflammation after MCAO. We then performed ELISA to examine the levels of the proinflammatory factors TNF‐a, interleukin 1 beta (IL1β), and IL6 in the brain tissue to test whether IU1 treatment inhibited neuroinflammation. The results showed that the protein expression of TNF‐a, IL‐1β, and IL‐6 was increased in the control mice but was reduced after IU1 treatment (Figure 4). Taken together, these results support the view that IU1 treatment inhibits the inflammatory cell response and attenuates neuroinflammation after MCAO.

### 3.4 IU1 reduced the infarct volume and neuronal loss after MCAO

**FIGURE 5:**
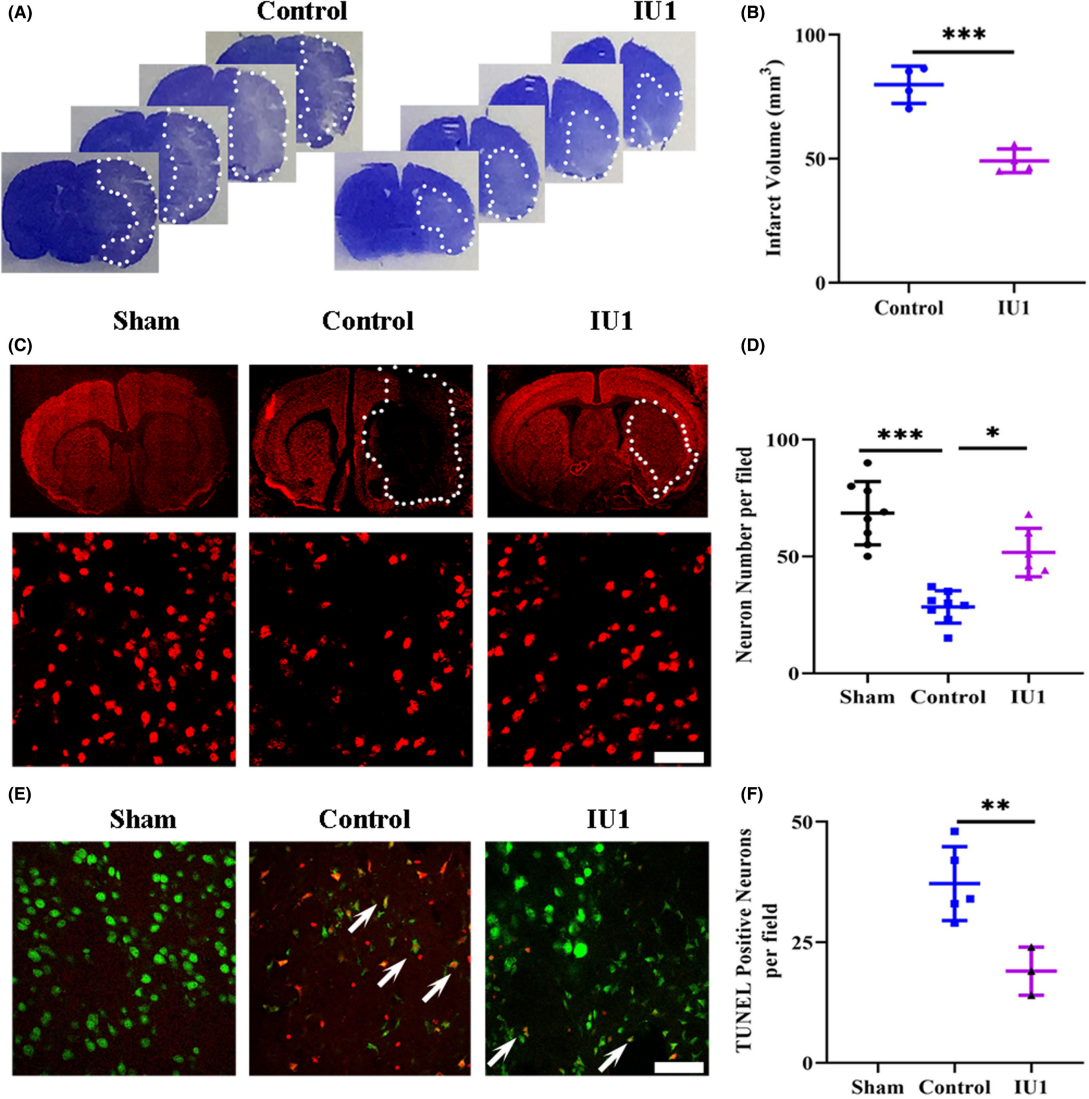
IU1 injection reduced neuronal loss 3 days after middle cerebral artery occlusion. (A) Typical images of crystal violet staining showing the infarct area in control and IU1‐treated mice. The dotted line indicates the border between infarcted tissue and noninfarcted tissue. (B) Bar graph showing the quantification of crystal violet staining; *n* = 4 in each group; ***p < 0.001 using two‐tailed unpaired *t* test. (C) Photo micrographs showing NeuN staining in the brain in each group. The upper panels show whole brain section images, and the lower panels show high‐magnification images; bar = 50 μm. (D) Bar graph showing the quantification of NeuN staining from the high‐magnification images; *n* = 8 in the sham group, *n* = 8 in the control group and *n* = 6 in the IU1 group; ***p < 0.001, *p < 0.05 using one‐way ANOVA followed by Bonferroni's post hoc test. (E) Typical images of TUNEL and NeuN double staining showing neuronal apoptosis in each group 3 days after MCAO. The arrowheads indicate TUNEL and NeuN double‐positive neurons; bar = 50 μm. (F) Histogram showing the quantification of neuronal apoptosis in the brain; *n* = 5 in the sham group, *n* = 5 in the control group and *n* = 3 in the IU1 group; **p < 0.01 using one‐way ANOVA followed by Bonferroni's post hoc test.

We used cresyl violet staining to determine the effect of IU1 on the infarct volume after MCAO. As shown in Figure 5A, MCAO induced large infarcts, including in the cortex and striatum. USP14 inhibition by IU1 greatly reduced the infarct volume in the MCAO group compared with the control group at 3 days after MCAO (Figure 5B, *p* = 0.0005). Then, we selected NeuN to label neurons to examine neuronal loss after MCAO. Consistent with the infarct volume results, neuronal loss was profoundly reduced by IU1 injection (Figure 5C,D, *p* = 0.0001 and *p* = 0.0176). Finally, we performed TUNEL staining, and the results showed that USP14 inhibition prevented neuronal apoptosis after MCAO (Figure 5E,F, *p* = 0.0001 and *p* = 0.0026). Taken together, these results suggest that USP14 inhibition reduces the infarct volume and prevents neuronal loss after MCAO.

### 3.5 IU1 reduced neurological deficits after MCAO

**FIGURE 6:**
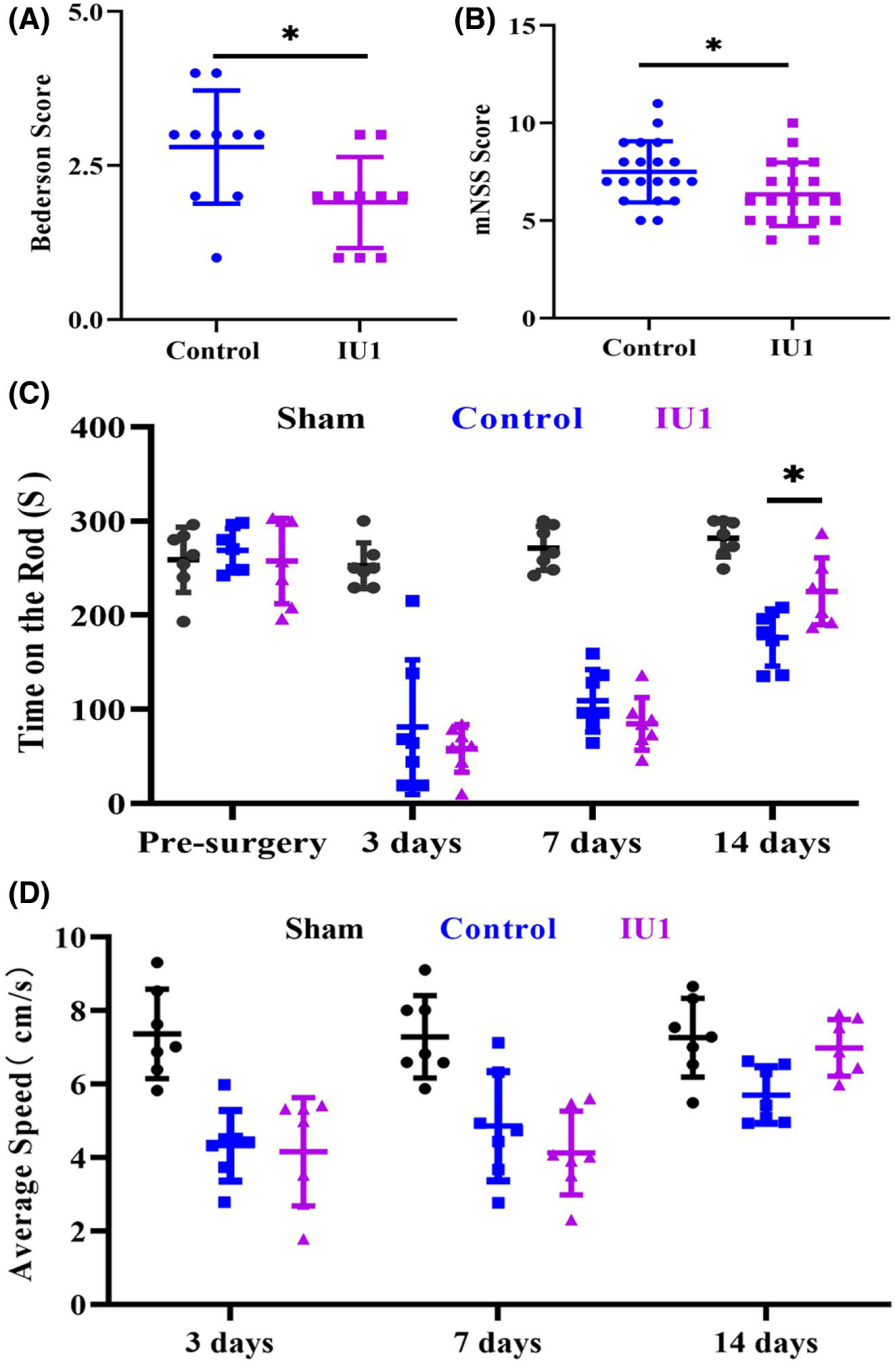
USP14 inhibition by IU1 promoted neurological function recovery after middle cerebral artery occlusion. (A) Bar graph showing Bederson scores; a higher score indicated more severe ischemic brain injury; *n* = 10 in each group; *p < 0.05 using the Mann–Whitney test. (B) Bar graph showing mNSSs; a higher score indicated more severe ischemic brain injury; *n* = 20 in each group; *p < 0.05 using two‐tailed unpaired *t* test. (C) Histogram showing performance in the rotarod test; *n* = 7–8 in each group; *p < 0.05 using two‐way ANOVA followed by Bonferroni's post hoc test. (D) Histogram showing performance in the open field test; *n* = 7–8 in each group.

Finally, we examined whether USP14 inhibition could impact neurological function in MCAO mice. First, we used modified Bederson scores to evaluate the effects of IU1 on ischemic stroke–induced acute neurological impairment. The results showed that the IU1‐treated mice had a lower score than the vehicle‐treated mice, suggesting the effect of IU1 in attenuating brain injury (Figure 6A, *p* = 0.0377). mNSSs, which were used as a composite of motor function, reflexes, and balance, displayed the same trend as Bederson scores (Figure 6B, *p* = 0.029). The latency to fall in the rotarod test, which was used to assess motor coordination and balance alterations, revealed that motor function recovered at 14 days after MCAO (Figure 6C, *p* = 0.0001 and *p* = 0.002). The average speed in the open field test, which was used to evaluate motor function and exploratory locomotor activity, was not significantly different after USP14 inhibition (Figure 6D). Together, these results support the role of IU1 in promoting motor function recovery in ischemic stroke mice.

## 4 DISCUSSION

In this study, we demonstrated the anti‐inflammatory effect of the USP14 inhibitor IU1 after ischemic stroke. Our results showed that ischemic stroke induced higher USP14 expression on endothelial cells and that IU1 injection could efficaciously attenuate ischemic brain injury and enhance neurological function. Furthermore, we showed that USP14 inhibition preserved BBB integrity and subsequently inhibited neuroinflammation by maintaining the expression of the tight junction protein ZO‐1, suggesting that it is a promising treatment approach for ischemic stroke.

Delayed clearance of misfolded proteins results in abnormal protein deposition, which leads to improper cell signal transduction and even cell death. In cells, protein degradation is mainly mediated by the UPS and the autophagy–lysosome pathway.^15^ While a large number of studies have explored the role of autophagy in recovery after ischemic stroke,^32^ the roles of the UPS in stroke recovery, especially the role of DUBs, are currently not well understood. As an important DUB, USP14 has been reported to be an important factor that negatively affects neuronal survival after ischemic injury. For example, injection of the USP14‐specific inhibitor IU1 attenuates neuronal damage resulting from ischemic stroke by directly reducing abnormal protein aggregation.^27^ Other studies have reported that downregulation of USP14 using miR‐124 or IU1 can inhibit the apoptosis of neurons and reduce the infarct volume by downregulating REST expression.^33^, ^34^ Recently, a study also showed that USP14 inhibition downregulated NCAO4 expression to prevent neuronal ferroptosis after ischemic stroke.^18^ More importantly, USP14 inhibition was found to increase the number of newborn neurons after MCAO.^33^ In our study, we found enhanced USP14 expression on endothelial cells 3 days after MCAO, and IU1 inhibition prevented BBB leakage and inhibited neuroinflammation, decreasing neuronal loss and favoring neurological function recovery. Our results expand our understanding of the role of USP14 in stroke pathology.

Endothelial cells form the first layer of the BBB to prevent substances from moving from the blood to the brain and thus represent a potential therapeutic target for neurological diseases. After ischemic stroke, typical endothelial tight junction proteins, such as ZO‐1, are degraded, leading to BBB dysfunction and a microenvironment that is detrimental to neuronal survival. Although studies have provided strong evidence for the degradation of tight junction proteins after ischemic stroke, the mechanism by which these proteins are degraded under these conditions is not fully understood. In addition to ROS and MMPs, other molecular mechanisms, such as the UPS and autophagy, which are typical protein degradation pathways, are responsible for the direct degradation of tight junction proteins.^14^, ^35^ Using an in vitro BBB model, Chen et al.^36^ found that Ubr‐1 upregulation activated the UPS, resulting in ZO‐1 degradation. Recently, autophagy was also shown to be involved in the degradation of tight junction proteins.^37^ In our study, increased USP14 expression in endothelial cells positively correlated with decreased ZO‐1 expression in MCAO mice, and these changes were reversed by direct USP14 inhibition. Further in vitro results showed significantly effects of IU1 in preventing ZO‐1 degradation. The results of the EB assay and IgG staining showed that USP14 inhibition effectively attenuated BBB leakage. Taken together, these results suggest that the USP14‐mediated protein degradation pathway may contribute to alterations in ZO‐1 levels and subsequent disruption of BBB integrity after ischemic stroke.

As the only USP family that can reversibly bind the 19S regulatory particle of the proteasome, USP14 plays a critical role in regulating the inflammation progression in various diseases, such as lung injury and atherosclerosis.^16^, ^38^ For example, Zhang et al.^20^ demonstrated that USP14 downregulation directly reduced proinflammatory cytokine release in mouse macrophage cell lines by inhibiting NF‐kb activation. However, the role of Ups14 in neuroinflammation under disease conditions is largely unknown. Inflammation progression occurs via different cellular mechanisms in the brain than in peripheral organs. After ischemic stroke, disruption of the connection between endothelial cells and/or astrocytes results in the infiltration of circulating immune cells from the blood into the brain. Both infiltrating cells from the blood and resident glial cells orchestrate the poststroke inflammatory response.^10^ In the current study, we demonstrated that USP14 inhibition through IU1 injection profoundly reduced the infiltration of neutrophils into the ischemic brain 3 days after MCAO. In addition to the number of inflammatory cells from the blood, the number of activated resident inflammatory glial cells, such as microglial cells and astrocytes, was markedly reduced after IU1 treatment. Furthermore, the release of cytokines from inflammatory cells was significantly decreased after USP14 inhibition. Based on these results, targeting USP14 may represent an effective approach to inhibit neuroinflammatory reactions after ischemic stroke.

In conclusion, our study demonstrates a role for USP14 in endothelial cells and BBB dysfunction as well as subsequent neuroinflammation after ischemic stroke, suggesting that targeting endothelial cell tight junction protein degradation through USP14 inhibition is an attractive therapeutic strategy for attenuating neuroinflammation and brain injury after ischemic stroke. Identification of this novel role of USP14 has implications for research on the pathology of ischemic stroke as well as other CNS diseases.

## AUTHOR CONTRIBUTIONS

Xiaosong He and Guo‐Yuan Yang supervised the entire project. Wenzhong Hou, Jianping Yao, Xiaohong Lin, and Xiaosong He designed most of the experiments, analyzed the data, and wrote the manuscript. Junjie Liu performed the injections and immunostaining. Juexian Wei and Xiaofan Chen contributed to the animal work. Hongbiao Huang provided IU1. Xiaohui Chen wrote part of the manuscript. All authors have reviewed the manuscript.

## CONFLICT OF INTEREST STATEMENT

None.

## Data Availability

The datasets generated and/or analyzed during the current study are available from the corresponding author on reasonable request.
